# Self-care and adherence to medication: a survey in the hypertension outpatient clinic

**DOI:** 10.1186/1472-6882-8-4

**Published:** 2008-02-08

**Authors:** Faekah Gohar, Sheila M Greenfield, D Gareth Beevers, Gregory YH Lip, Kate Jolly

**Affiliations:** 1 Medical Student, University of Birmingham, Birmingham, B15 2TT, UK; 2 Dept of Primary Care, University of Birmingham, Birmingham, B15 2TT, UK; 3 University Department of Medicine at City Hospital, Birmingham B18 7QH. UK; 4 Dept Public Health, University of Birmingham, Birmingham, B15 2TT, UK

## Abstract

**Background:**

Self-care practices for patients with hypertension include adherence to medication, use of blood pressure self-monitoring and use of complementary and alternative therapies (CAM) The prevalence of CAM use and blood pressure self-monitoring have not been described in a UK secondary care population of patients with hypertension and their impact on adherence to medication has not been described. Adherence to medication is important for blood pressure control, but poor adherence is common. The study aimed to determine the prevalence of self-care behaviours in patients attending a secondary care hypertension clinic.

**Methods:**

Cross-sectional questionnaire survey. 196 patients attending a secondary care hypertension clinic in a teaching hospital serving a multiethnic population, Birmingham, UK. Main outcome measures: Prevalence of use of CAM, home monitors, adherence to anti-hypertensive medication.

**Results:**

CAM use in previous 12 months was reported by 66 (43.1%) respondents. CAM users did not differ statistically from non-CAM users by age, gender, marital status or education. Vitamins, prayer a dietary supplements were the most commonly used CAM. Nine (12.7%) women reported using herbal CAM compared to one man (1.2%), (p = 0.006). Ten (6.7%) respondents reported ever being asked by a doctor about CAM use. Perfect adherence to anti-hypertensive medication was reported by 26 (44.8%) CAM-users and 46 (60.5%) non-CAM users (p = 0.07). Being female and a CAM user was significantly associated with imperfect adherence to anti-hypertensive medication. Older and white British respondents were significantly more likely to report perfect adherence. Blood pressure monitors were used by 67 (43.8%) respondents, which was not associated with gender, CAM use or adherence to medication.

**Conclusion:**

Hypertensive patients use a variety of self-care methods, including CAM, home blood pressure monitors, and adherence to prescribed medication. This study found the prevalence of CAM use in hypertensive patients was higher than in the UK population. It is important to acknowledge the self-care behaviour of hypertensive patients, in order to assess potential harm, and encourage effective methods of self-care.

## Background

Self-care describes the maintenance of health, prevention and treatment of illness by an individual [1,2]. It has been shown to improve patient satisfaction, quality of life, and also reduce primary care, outpatient and emergency department visits [3]. The four components of self-care are leading a healthy lifestyle, treating minor ailments, managing chronic conditions and care after hospital discharge [1]. The National Health Service in the UK is committed to supporting and making provision for self-care in the management of chronic conditions [2], which can include empowering patients to self-manage their condition using home blood pressure monitors, as well as encouraging adherence to medication [3]. Hypertension (high blood pressure) is a highly prevalent chronic condition, affecting 32% of men and 30% of women aged 16 or over in England [4]. It is a modifiable but important risk factor for coronary heart disease and stroke [5]. Self-care for hypertension includes lifestyle measures such as maintaining a healthy weight and diet, as well as adhering to medication, to help patients achieve blood pressure (BP) control [3,5,6].

One form of self-care, complementary and alternative medicine (CAM) is becoming increasingly popular, with overall CAM sales in the UK rising by 23% since 1998 [7]. Although the one-year prevalence of CAM use in the general UK population is 20% [7], a higher prevalence of CAM use has been associated with a diagnosis of chronic disease [8-10]. There have been no reports of CAM use in hypertensive patients attending secondary care clinics in the UK, but CAM use in hypertensives has been reported for the USA [10,11] and in Singapore [12] and other studies have focussed on cardiovascular disease [13-15]. In addition, the evidence base for using CAM to manage hypertension has not been demonstrated definitively, as studies assessing the influence of CAM on BP reduction have been small scale, or uncontrolled [16], although CAM relaxation therapies have been reported to produce small BP improvements [17].

Supplements or natural products, containing for example herbs, can cause unpredicted effects, drug interactions, and even compromise BP control [5,18,19]. However it is known that patients do not limit their CAM use to conditions where its use is evidence-based [9], or proven to be safe [15]. There is also evidence that CAM users fail to report their use of CAM [7,18] and are more likely than non-CAM users to believe CAM is safe [20,21]. It is therefore imperative to have specific information on the prevalence of CAM use in the hypertensive clinic.

Poor adherence to prescribed medication is associated with treatment failure and poor BP control [22,23], with only 50–70% of hypertensive patients adherent, and the prevalence of adherence varying by the study population, length of diagnosis and method of assessment [22]. Up to 50% of CAM users in the general population report using CAM alongside prescribed medication [18], as CAM tends to be used as a complement rather than as a replacement for conventional medicine [9,21], this should not adversely affect adherence to prescribed anti-hypertensive medication. However, CAM use is decided by many factors, which may be the positive qualities of CAM, or negative aspects of conventional care [24]. Although adherence has been extensively researched, it is not fully understood [22], and as CAM prevalence increases, its influence on adherence requires consideration. Current research into the influence of CAM on adherence is limited and has not focussed on hypertensive patients.

Like the use of CAM and adherence to prescribed medication, the use of home blood pressure monitors is another aspect of self-care relevant to the management of hypertension. The use of home monitoring BP devices can aid diagnosis, has been shown to be effective in improving BP control, although the mechanism for this is unknown [25], and also reduces the 'white coat effect' [23,26,27]. The influence of home BP monitors on adherence to anti-hypertensive medication is less well known [28]. Like CAM, there has been recent interest from the public in purchasing home monitors from pharmacies and over the internet [23], and the only published survey of use of BP self-monitoring reported a 9% prevalence in a postal survey of a UK adult primary care population [29]. Logistic regression showed predictors of use were increasing age, female gender, having a university degree and living in a more affluent area

Home BP monitors have a role in encouraging personal control of hypertension and other cardiovascular disease risk factors [26] and can positively reinforce self-care behaviour such as adherence to medication in the management of chronic disease [30].

The objectives of the study reported here were to describe (i) the prevalence of and factors associated with CAM use in hypertensive patients attending secondary hypertension clinics, (ii) adherence to anti-hypertensive medication and its relationship to CAM use, and (iii) the use of home blood pressure monitors by hypertensive patients, as part of self-care.

## Methods

This cross-sectional descriptive survey was undertaken at outpatient hypertension clinics at a teaching hospital in Birmingham, UK. This hospital serves a multiethnic community of 350,000, with an ethnic prevalence of approximately 25% south Asian, 11% Afro-Caribbean and 64% white European [31]. Between February and April 2006, 196 consecutive English speaking or non-English speaking patients with family members able to translate, were approached and asked to complete a questionnaire by the researcher. Written consent was obtained. The questionnaire was verbally administered to consenting respondents who were unable to self-complete the survey. Ethical approval was granted by the local Research Ethics Committee.

### Survey design

Data were collected on demographic characteristics, CAM use, adherence to prescribed medication and use of home blood pressure monitors. CAM use over the previous 12 months was identified using a tick list based on the five categories of CAM classified by the National Centre for Complementary and Alternative Medicine [32]. One-year prevalence, rather than lifetime use, was used to reduce recall bias [33]. The following open question asked responders to specify which dietary supplements or vitamins were used: 'if you have ticked dietary supplements or vitamins, please list the names of all those which you have used (e.g. vitamin C, garlic capsules, cod liver oil, Sanatogen etc). The use of CAM specific for the management of hypertension was not determined. Instead, responders were asked about any CAM use over the past year, as it was felt any CAM use, whether or not specifically used for hypertension, could interact with medication [7]. In addition, any CAM use could influence adherence to anti-hypertensive medication, as it has been shown that CAM users are often dissatisfied with conventional care [7]. Respondents were also asked if they had ever been asked by a doctor about CAM use.

Adherence to prescribed medication was tested using an adapted version of the Hill-Bone compliance to high blood pressure therapy scale [34,35]. Using 8 questions, this assessed adherence by assessing patients' forgetfulness in taking medication, filling prescriptions and ensuring they did not run out of medication and asked about circumstances in which patients might not take their BP medication. Minor wording changes were made to modify the American scale for use in the UK setting [see additional file 1]. Responses ranged from none of the time (scoring 1) to some, most, or all of the time (scoring 4), so adherence scores of 8 and greater than 8 indicated perfect and imperfect adherence respectively.

### Statistical analysis

SPSS version 13.0 was used for data analysis. Chi-squared and Fishers Exact test, as appropriate, were used to analyse categorical variables, comparing gender, ethnicity and marital status with CAM use, home blood pressure monitoring equipment use and adherence to medication (perfect or imperfect). CAM users were defined as those who had used at least one CAM in the previous 12 months. Logistic regression was used for the univariate analysis of perfect adherence, CAM use and use of home BP monitoring tests with the continuous variables age, education and time since their diagnosis of hypertension. Tests were 2-tailed, and a p-value ≤ 0.05 was considered statistically significant. A multivariate analysis of the predictors of perfect adherence to prescribed medication was undertaken.

## Results

### Characteristics of sample

Of 196 questionnaires distributed, 153 (78.8%) were completed. The sample included similar numbers of male and female respondents, with 82 (53.6%) being male and mean age 57.3 (SD = 16.0) years (Table 1).

**Table 1:** Characteristics of respondents: users and non-users of CAM

	**Total (100%) n = 153**	**CAM users (43.8%) n = 67**	**Non-CAM users (56.2%) n = 86**
Mean age, years (SD)	57.3 (16.0)	55.9 (15.7)	58.4 (16.3)
Age < 60 years	79 (51.6)	39 (58.2)	40 (46.5)
Gender			
males	82 (53.6)	34 (50.7)	48 (57.0)
females	71 (46.4)	33 (49.3)	38 (44.2)
Marital Status			
Single	31 (20.3)	14 (20.8)	17 (19.8)
Married/co-habiting	89 (58.2)	34 (50.7)	55 (64.0)
Divorced/separated	15 (9.8)	11 (16.4)	4 (4.7)
Widowed	17 (11.1)	7 (10.4)	10 (11.6)
Mean years in Education (SD)	12.25 (4.5)	12.20 (4.0)	12.28 (4.9)
Ethnicity (%)			
White British	90 (59.2)	33 (49.3)	57 (66.3)
White Irish	7 (4.6)	6 (9.0)	1 (1.7)
Asian/Asian British Indian	18 (11.8)	10 (14.9)	8 (9.3)
Asian/Asian British Pakistani	9 (5.9)	3 (4.5)	6 (7.0)
Black/Black British Caribbean	19 (12.5)	12 (17.9)	7 (8.1)
Other^1^	9 (6.1)	2 (3.0)	7 (8.1)
Co morbidity			
Poor circulation in legs	41 (26.8)	21 (31.3)	20 (23.3)
Diabetes	24 (15.7)	11 (16.4)	13 (15.1)
Stroke	16 (10.5)	8 (11.9)	8 (9.3)
Angina	20 (13.1)	11 (16.4)	9 (10.5)
Heart valve problems	12 (7.8)	10 (14.9)	2 (2.3)
Heart attack	8 (5.2)	4 (8.0)	4 (4.7)
Atrial fibrillation	7 (4.6)	2 (3.0)	5 (5.8)
Renal complications	7 (4.6)	6 (9.0)	1 (1.2)
Asthma	6 (3.9)	2 (3.0)	4 (4.7)
Arthritis/musculoskeletal	13 (8.5)	8 (11.9)	5 (5.8)
Hypertension			
Mean duration of hypertension diagnosis, years	10.1 (SD = 10.2)	9.2 (SD = 7.8)	10.9 (SD = 11.9)
Hypertensives diagnosed within last year (%)	17 (11.1)	7 (10.4)	10 (11.6)

^1^Other ethnicity: White other (n = 3); Asian/Asian British Bangladeshi (n = 1); Asian/Asian British other (n = 1); Black/Black British African (n = 3); Mixed (n = 1).

### Assessment of CAM use

CAM use was organised and analysed according to the five categories identified by the National Centre for Complementary and Alternative Medicine: alternative medical; mind-body; manipulation/body-based; energy, and biological based therapies. CAM users did not differ significantly from non-CAM users by age, gender, marital status, education, ethnicity or comorbidity. Sixty-seven (43.7%) respondents had used at least one CAM in the past 12 months. Excluding prayer, the CAM prevalence was 37.9% (n = 58). Vitamin supplements, prayer, dietary supplements and relaxation were the most frequently used (Table 2). Cod liver oil, multi-vitamins and garlic were the most popular vitamin and dietary supplements. Nine (12.7%) of women reported using herbal CAM compared to one man (1.2%), (p = 0.006). Although more women used CAM than men (33, 49.3% v 33, 40.2%), this was not significant. Only 10 (6.7%) respondents reported ever being asked by a doctor about their use of CAM.

**Table 2:** Use of CAM, vitamin & dietary supplements, by gender

	**Males (%) n = 82**	**Females (%) n = 71**	**Total (%) n = 153**
**Used CAM**^**1**^	33 (40.2)	33 (49.3)	66 (43.1)
Excluding Prayer	28 (34.1)	30 (42.3)	58 (37.9)
**Alternative medical system**			
Chinese medicine	2 (2.4)	2 (2.8)	4 (2.6)
Homeopathy	0	3 (4.2)	3 (2.0)
**Mind-body intervention**			
Prayer	11 (13.4)	8 (11.3)	19 (12.4)
Relaxation	6 (7.3)	7 (9.9)	13 (8.5)
Aromatherapy	3 (3.7)	4 (5.6)	7 (4.6)
Meditation	1 (1.2)	3 (4.2)	4 (2.6)
Spiritual healing	0	3 (4.2)	3 (2.0)
Yoga	0	2 (2.8)	2 (1.3)
Tai chi	0	1 (1.4)	1 (0.7)
**Manipulative/body-based**			
Massage therapy	4 (4.9)	3 (4.2)	7 (4.6)
Acupuncture	3 (3.7)	2 (2.8)	5 (3.3)
Reflexology	1 (1.2)	3 (4.2)	4 (2.6)
**Energy therapies**			
Energy healing	0	2 (2.8)	2 (1.3)
Magnetic therapy	1 (1.2)	0	1 (0.7)
**Biological Based**			
Vitamin supplements	14 (17.1)	17 (23.9)	31 (20.3)
Dietary supplements	11 (13.4)	6 (8.5)	17 (11.1)
Herbal medicine^3^	1 (1.2)*	9 (12.7)*	10 (6.5)
Any biological therapy	19 (23.2)	25 (35.2)	44 (28.8)
**Vitamin/Dietary Supplements**^**2**^			
Cod liver oil	11 (13.4)	10 (14.1)	21 (13.7)
Multivitamins	4 (4.9)	7 (9.9)	11 (7.1)
Garlic	3 (3.7)	5 (7.0)	8 (5.2)
Vitamin C	3 (3.7)	4 (5.6)	7 (4.6)
Glucosamine	3 (3.7)	3 (4.2)	6 (3.9)
Vitamin B/B12	2 (2.4)	1 (1.4)	3 (2.0)
Omega-3	1 (1.2)	1 (1.4)	2 (1.3)
Nutritional drinks	0	2 (2.8)	2 (1.3)
Primrose Oil	0	2 (2.8)	2 (1.3)

^1^CAM Modalities not used: Ayurveda, Naturopathy, Qi Gong (Alternative system), Biofeedback, Guided Imagery, Hypnosis (Mind-body interventions), Chelation (Biological), and chiropractic (Manipulative technique).

^2^Vitamin/diet supplements used by n = 1 (0.7%): Vitamin D, Folic Acid, Cranberry Juice, Selenium, Spirulina, Chondroitin, Silica.

^3^Significant *p *= 0.006.

^4^Vitamin & dietary supplements were analysed together

### Assessment of adherence to anti-hypertensive medication

134 (87.6%) respondents reported they were prescribed anti-hypertensive medication. Twenty six (44.8%) users compared to 46 (60.5%) non-users of CAM who reported being prescribed antihypertensive medication reported perfect adherence (Table 3). No significant differences were observed between patients who reported perfect or sub-optimal medication adherence for gender, any CAM use, CAM use excluding vitamin and dietary supplement use, ethnicity, duration since hypertension diagnosis, or education. However, a significant difference between female CAM and non-CAM users and adherence was found, with eleven (35.9%) females using CAM reporting perfect adherence, compared to 23 (63.9%) women who had not used any CAM in the previous twelve months (p = 0.02). The same analysis was not significant for males.

**Table 3:** Adherence to anti-hypertensive medication

	**Perfect Adherence (%)**	**Non-perfect Adherence (%)**	**Total (%)**	**p value**
Gender				
Males	38 (56.7)	29 (43.3)	67	0.49
Females	34 (50.7)	33 (49.3)	67	
Cam users				
Cam use	26 (45.6)	31 (54.4)	57	
No CAM use	46 (59.7)	31 (40.3)	77	0.11
Gender and CAM use				
Male & CAM	15 (57.7)	11 (42.3)	26	
Male & no CAM	23 (56.1)	18 (43.9)	41	0.90
Female & CAM	11 (35.5)	20 (64.5)	31	
Female & no CAM	23 (63.9)	13 (36.1)	36	0.02
Ethnicity				
White British	55 (67.1)	27 (32.9)	82	<0.001
All other	17 (32.7)	35 (67.3)	52	
Age (SD)	60.17 (14.7)	53.72 (16.2)	56.95 (15.5)	0.02
BP monitor				
Users	29 (49.2)	30 (50.8)	59	0.55
Non-users	33 (44.0)	42 (56.0)	75	
Duration of hypertension diagnosis, yr (SD)	9.96 (10.54)	11.30 (10.9)	10.63 (10.72)	0.48
Education, yr (SD)	12.44 (4.70)	12.32 (4.14)	12.38 (4.42)	0.88

Pearson's chi-squared showed white British respondents were significantly more likely to be perfectly adherent compared to all other ethnicities (67.1% v 32.9%, p < 0.001). The mean age of adherent patients was 60.2 years (range 23 to 86 years) compared to 53.7 years (range 23 to 88 years) for non-adherent patients (p = 0.02). In a logistic regression model including age, gender, ethnicity, CAM use, duration of hypertension and years of education, increasing age predicted perfect adherence (OR 1.03, 95% CI 1.00, 1.06) and being of ethnic minority ethnicity predicted lower adherence (OR 0.31, (95% CI 0.14, 0.72).

### Assessment of use of home blood pressure monitors

Home BP monitors were used by 66 (43.1%) of respondents (Table 4). The main source of monitors was pharmacies and chemists for 30 (45.5%) respondents, and 15 (22.7%) obtained them from family or friends. Twenty-seven (40.9%) BP monitor users reported using the monitor for monitoring purposes, while 8 (12.1%) had used a monitor after a high reading, in order to diagnose hypertension. The use of BP monitors was not significantly associated with gender, any CAM use, CAM use excluding vitamin and dietary supplement use, or adherence to anti-hypertensive medication.

**Table 4:** Use of home monitoring tests for all respondents, and by CAM use and gender

	Total (%) n = 153	CAM users (%) n = 67	No CAM (%) n = 86
**BP home monitor**	66 (43.8)	33 (50.7)	33 (38.4)
**Reason for home BP monitor**			
hypertension diagnosis	8 (14.3)	5 (15.2)	3 (9.1)
advised by doctor	7 (12.5)	3 (9.1)	4 (12.1)
to aid diagnosis	4 (7.1)	3 (9.1)	1 (3.0)
felt unwell/concerned	4 (7.1)	1 (3.0)	3 (9.1)
for monitoring	27 (48.2)	15 (45.5)	12 (36.4)
already had access	2 (3.6)	1 (3.0)	1 (3.0)
curiosity	1 (1.8)	0	1 (3.0)
can't remember	1 (1.8)	0	1(3.0)
**Source of monitor**			
Pharmacy/chemist	30 (45.5)	16 (48.5)	14 (42.4)
Postal ordered	6 (9.1)	3 (9.1)	3 (9.1)
Internet	1 (1.5)	0	1 (3.0)
Family/friend	15 (22.7)	8 (24.2)	7 (21.2)
Doctor	5 (7.6)	3 (9.1)	2 (6.1)
Other	9 (13.6)	3 (9.1)	6 (18.2)

BP: blood pressure

## Discussion

### Prevalence of CAM use

A higher prevalence of CAM use in this secondary care hypertensive population was found compared to the reported UK general population, reflecting findings of studies of other chronic disease patients [8,36,37]. Eisenberg et al. showed that despite a high prevalence of use of CAM by hypertensive patients also using conventional care, none believed that CAM was better than conventional care for their condition [38].

A CAM prevalence (excluding non-prescribed vitamin or dietary supplement use) of 14.5% in diagnosed hypertensives was described by a primary care study in Singapore [12], which is lower than the comparative figure in our study (26.1%). Surveys of CAM use in cardiovascular disease in the US have found a greater prevalence of use than is seen in the general population, which is itself higher than in the UK [8,10,11]. A survey of pre-cardiac surgery patients which included exercise as a CAM modality [10] found a CAM prevalence of 80.9% and a large population survey in the USA reported a lower prevalence of 69.5% of hypertensives aged 65 years or more [11]. A survey in an American cardiovascular outpatient clinic of biological-based CAM therapies found a one-year prevalence of 42% [15], compared to 28.8% found in this study. These studies are not directly comparable with this research due to differences in range of cardiovascular diagnoses included, the study setting and the CAM categories included.

Population studies have shown CAM use to be significantly associated with being white, middle aged, well educated and female [11,21,37,39]. However, no significant differences between CAM and non-CAM users for ethnicity, age, education or gender were reported in our sample of hypertensive patients. Within CAM use, there were also no significant differences by gender, other than for herbalism, which was significantly more likely to be used by females. These findings suggest CAM users in the hypertension clinic do not fit the demographic patterns seen in population studies. However, the definitions of CAM use vary between surveys which may also explain the differences found. In addition, our survey was carried out at one UK teaching hospital, and therefore are not generalisable to all hypertensive patients attending secondary care outpatient clinics. Clinic non-attendees were obviously excluded, and 21% of patients approached did not complete a questionnaire, introducing some response bias.

CAM use in ethnic minorities has been little established, although patterns of CAM use have been shown to differ by ethnicity [39,40]. In our study, white British and Asian British Pakistani respondents were least likely to report CAM use. The use of CAM by black and Asian adults was also indicated to be considerable in a US population study [8]. There is therefore a complex association between ethnicity and CAM use which requires further investigation, as, although this sample was ethnically diverse, the numbers in individual ethnic groups were too small to allow significant conclusions to be made. One limitation of our study was the inclusion of questionnaires translated by family members, rather than the ideal of either a translated questionnaire or use of an interpreter. Although less than 5% of the sample, these were included in order to avoid exclusion bias. It was also felt that this mirrored the situation in the consultation itself, and that the questionnaire did not cover particularly sensitive issues which would require an independent interpreter. We acknowledge that the relatively small size of our sample is a limitation of our study.

The biological therapies, vitamin, dietary and herbal supplements, were the most used CAM group, and within these, cod liver oil and garlic were among the three most used supplements. Cod liver oil contains omega-3 fatty acids, the benefits of which are still unproven for cardiovascular disease [14]. The high doses required for BP reduction have significant side effects and as a result omega-3 is not routinely recommended as a treatment for hypertension [6]. The benefits of garlic supplementation also remain unproven, with many small trials reporting only short-term BP improvements [7,18].

Vitamins and dietary supplements were included in this survey as biological-based CAM therapies as they are widely accessible, available over the counter, and have been shown to have a high prevalence of use in population surveys [8,18,41]. Prayer was also included in this study as a result of its inclusion in large CAM prevalence surveys [8,11,21]. The definition of CAM is wide [32,42] which explains why surveys vary in the number and categorization of CAM therapies included [42]. To explore the possibility that our findings may have been strongly influenced by the inclusion of vitamins and dietary supplements, we re-ran the analyses using a narrower definition of CAMs (excluding vitamins and dietary supplements). Our findings did not change in relation to adherence to antihypertensive medication and use of home BP monitors.

After vitamins, dietary supplements and prayer use, the most frequently used CAM therapies were relaxation, herbal medicine, aromatherapy and massage therapy. These are amongst the most commonly used in the UK population, along with acupuncture, reflexology, and homeopathy [36]. Studies are ongoing into the benefits of CAM for hypertension, but relaxation therapies are not recommended currently as part of routine NHS treatment for hypertension [5].

### Reporting CAM use to a medical practitioner

Only 6.7% of the sample reported being asked about their CAM use by a doctor which compares to a previously reported figure of 3.4% [8]. Knowledgeable doctors trained in CAM are more likely to ask about CAM use [43,44]. However, a study in Israel reported that doctors estimated that 15% of their patients used a CAM, suggesting that they underestimate their patients' CAM use [43], and found that many were unaware of the potential harms of these therapies [43,44]. Patients are also reluctant to voluntarily disclose their use of CAM, with 57% to 70% failing to tell their doctor about CAM use [18,20,21,38] with fear of disinterest by the treating physician being the most commonly cited reason for this [45]. Only a minority of patients use CAM prior to accessing conventional services so this could be addressed by broaching the issue of CAM use sensitively, avoiding making assumptions, while respecting patients' self-care choices and providing guidance regarding CAM and self-care [42,45].

### CAM use and adherence to antihypertensive medication

This study shows CAM use was significantly associated with reduced adherence to anti-hypertensive medication for females. The reason for this, and why females may differ from males in adherence to medication while using CAM requires further work. Studies evaluating the influence of CAM use on adherence are limited with conflicting results. Jernewall et al's study of HIV positive Latino men, found that use of plant-based CAM was significantly associated with non-adherence to conventional medication [46]. In contrast, a study of lung transplant patients found 88% were using some form of CAM, and CAM users did not differ from non-CAM users in the percentage reporting adherence to the transplant regime [47]. Further work is therefore required into the relationship between CAM use and adherence to prescribed medication before conclusions can be made.

In this study, seventy two (41.7%) respondents reported perfect adherence, which is lower than the 50 to 70% reported in previous studies of patients diagnosed with hypertension for more than one year [48]. This may be due to differences in the method of measuring adherence. This study also found older age and white British ethnicity to be positively related to perfect adherence, in line with previous research [48]. The gold standard for measuring adherence, electronic measurement, was an unsuitable method for this survey [23]. To reduce non-response and social acceptability bias, a confidentiality reminder clause was inserted immediately above the adherence question in the questionnaire and on the patient information sheet before consent was obtained.

While over 40% of the sample had used a home BP monitor, only a minority cited doctors' advice as the reason for its use. Pharmacies and chemists were the main source for monitors in this study, and retail sales are expected to increase further as self-care becomes more popular [23]. Home monitoring in a randomised control trial was shown to lead to less intensive medication regimes, but also resulted in reduced BP control [27]. Self-monitoring BP has also been shown to assist weight loss, reduce BP, to be cost effective and does not increase patient anxiety [26]. However, patients require training in using monitors, and understanding and interpreting readings appropriately to maximise the benefits of self-monitoring [23]. This was reflected by a systematic review which found that in six of the eleven randomised control trials included, adherence was significantly improved with home BP monitoring [28]. When home BP monitoring was used with other interventions, including patient counselling and education, the effect on adherence was greater [28]. The potential benefits should be recognised and further researched [26,27,49], and doctors need to be aware of the high level of patient use and routinely ask about this in the consultation.

## Conclusion

This study has shown hypertensive patients use self-care in a variety of forms, which include CAM and home monitors, as well as adhering to prescribed medication. The prevalence of CAM use in this study is higher than in the UK population. However not all the CAM methods used by the respondents have been proven to have beneficial effects by conclusive evidence-based research, and may even be potentially harmful. It is therefore important to monitor and encourage effective methods of self-care in association with proven conventional care.

A significant association between CAM use and less than perfect adherence was found for females in this study. CAM use may therefore have some influence on adherence, however this requires further investigation, as research is limited in this area.

Home blood pressure monitors were used by a significant proportion of this hypertensive sample in order to self-manage their condition, and only a minority of these patients had been recommended to use a BP monitor by a health care professional. Home BP monitoring is popular with patients, but patient education and training is required to achieve maximum benefits from the use of home monitors, and the role of monitors in the management of hypertension therefore requires further research.

## Competing interests

The author(s) declare that they have no competing interests.

## Authors' contributions

All authors conceived and designed the study. FG collected the data and undertook the analysis with support from KJ and SG. FG drafted the manuscript. All authors critically reviewed the manuscript, read and approved the final manuscript.

## Pre-publication history

The pre-publication history for this paper can be accessed here:



## Supplementary Material

Additional file 1 Questions adapted from the Hill-Bone Scale. The questionnaire used to assess adherence to medication, which was adapted from the Hill-Bone Scale. Click here for file
