# Supplementary material for: Self-care and adherence to medication: a survey in the hypertension outpatient clinic
Source: BMC Complement Altern Med. 2008 Feb 8;8:4. doi: 10.1186/1472-6882-8-4 (PMC2259297; doi:10.1186/1472-6882-8-4)
Supplement: Additional file 1 — Questions adapted from the Hill-Bone Scale. The questionnaire used to assess adherence to medication, which was adapted from the Hill-Bone Scale. [file 1472-6882-8-4-S1.doc]

# Additional file 1

Questions adapted from the Hill-Bone Scale

1. How often do you forget to take your blood pressure medication
2. How often do you decide not to take your blood pressure medication?
3. How often do you forget to get prescriptions filled?
4. How often do you run out of blood pressure medication?
5. How often do you not take your blood pressure medication before you go to the doctor?
6. How often do you not take your blood pressure medication when you feel better?
7. How often do you **miss taking** your blood pressure medication when you feel **unwell**?
8. How often do you take someone else’s blood pressure medication?

**Notes:**

Of the 14 measures of the full Hill-Bone Scale, only the 8 relating to medication use were used.

‘HBP’ was changed to blood pressure medication.

Other changes include substituting ‘miss taking’ for ‘skip’ and ‘unwell’ for ‘sick’ in the original scale, as highlighted above.
